# Estimating the cardiac signals of chimpanzees using a digital camera: validation and application of a novel non-invasive method for primate research

**DOI:** 10.3758/s13428-023-02136-y

**Published:** 2023-05-30

**Authors:** Danyi Wang, Johanna Eckert, Sam Teague, Ali Al-Naji, Daniel Haun, Javaan Chahl

**Affiliations:** 1 UniSA STEM, University of South Australia, Mawson Lakes, SA 5095 Australia; 2 Comparative Cultural Psychology, Max Planck Institute for Evolutionary Anthropology, Deutscher Platz 6, 04103 Leipzig, Germany; 3 Electrical Engineering Technical College, Middle Technical University, Baghdad, 10022 Iraq; 4 Leipzig Research Center for Early Child Development, Leipzig University, Jahnallee 59, 04109 Leipzig, Germany; 5 Platforms Division, Defence Science and Technology Group, Edinburgh, SA 5111 Australia

**Keywords:** Heart rate, Signal processing, Comparative psychology, Contact-free

## Abstract

Cardiac measures such as heart rate measurements are important indicators of both physiological and psychological states. However, despite their extraordinary potential, their use is restricted in comparative psychology because traditionally cardiac measures involved the attachment of sensors to the participant’s body, which, in the case of undomesticated animals such as nonhuman primates, is usually only possible during anesthesia or after extensive training. Here, we validate and apply a camera-based system that enables contact-free detection of animals’ heart rates. The system automatically detects and estimates the cardiac signals from cyclic change in the hue of the facial area of a chimpanzee. In Study 1, we recorded the heart rate of chimpanzees using the new technology, while simultaneously measuring heart rate using classic PPG (photoplethysmography) finger sensors. We found that both methods were in good agreement. In Study 2, we applied our new method to measure chimpanzees’ heart rate in response to seeing different types of video scenes (groupmates in an agonistic interaction, conspecific strangers feeding, nature videos, etc.). Heart rates changed during video presentation, depending on the video content: Agonistic interactions and conspecific strangers feeding lead to accelerated heart rate relative to baseline, indicating increased emotional arousal. Nature videos lead to decelerated heart rate relative to baseline, indicating a relaxing effect or heightened attention caused by these stimuli. Our results show that the new contact-free technology can reliably assess the heart rate of unrestrained chimpanzees, and most likely other primates. Furthermore, our technique opens up new avenues of research within comparative psychology and facilitates the health management of captive individuals.

**Supplementary Information:**

The online version contains supplementary material available at 10.3758/s13428-023-02136-y.

## Introduction

Cardiac signals are widely used as one of the main physical health parameters of both human and nonhuman individuals (e.g., Aiello, 2016; Hajar, 2018; Jensen, 2019). However, heart rate also plays a crucial role in psychological research. Already in the middle of the last century, it was found that emotionally arousing stimuli lead to measurable changes in heart rate in human adults (Darrow, 1929; Graham & Clifton, 1966; Lacey, 1959). That is, a participant’s heart rate can be indicative of their psychological activity. Especially in developmental psychology, measuring heart rate has proven an extremely useful tool to implicitly assess psychological activity in young participants who cannot yet verbally articulate their mental states or demonstrate complex behaviors (see Reynolds & Richards, 2008, for a review). Early research found, for example, that newborn infants’ heart rate accelerated when they experienced tactile or auditory stimulation (Davis et al., 1965). By contrast, heart rate decelerated as part of the orienting response, i.e., when infants focused their attention on a novel stimulus (e.g., Graham et al., 1983; Graham & Jackson, 1970). Indeed, heart rate can be used as an indicator for different phases of attention and information processing in infants. It has therefore been utilized in studies testing recognition memory, i.e., studies that tried to identify whether an infant perceived a stimulus as novel or as the same as a previously presented one. This has been done in the context of object categorization (Elsner et al., 2006), as well as in the context of numerical cognition (Brez & Colombo, 2012). Moreover, changes in heart rate have been used as a measure of positive affect in infants (Brock et al., 1986) and as a measure of infants’ ability to recognize the incongruity of absurd scenes in the context of humor perception (Mireault et al., 2018). In sum, measuring heart rate provides a fruitful way to assess both emotional states and cognitive processes in pre-verbal infants.

Measuring psychological activity in an implicit, non-verbal way is not only valuable for pre-verbal but even more so for non-verbal creatures. So far, however, only very few studies have implemented cardiac measures in cognitive research with animals, especially with humans’ closest living relatives, the nonhuman primates. These studies have mainly focused on heart rate as a measure of affective state. For chimpanzees (*Pan troglodytes*), for example, it was found that heart rate changes in response to hearing emotionally arousing auditory stimuli (Berntson et al., 1989) or viewing emotionally arousing pictures [(Boysen & Berntson, 1986; Boysen & Berntson, 1989); also see (Kano et al., 2016)]. Aureli et al. (1999) used heart rate as an indicator of emotional state during social interactions in rhesus macaques.

The relative dearth of studies using cardiac measures in comparative research is due to crucial practical limitations: until recently, measuring cardiac signals required one or several sensors to be attached to the participant’s body. In the case of nonhuman primates, this constitutes a severe constraint. This limitation implied that animals had to be trained extensively to tolerate the attachment of sensors, (e.g., Berntson et al., 1989; Boysen & Berntson, 1986; Boysen & Berntson, 1989; Kano et al., 2016; but see Cloutier Barbour et al., 2020), limiting the number of testable individuals to only a handful of animals worldwide. Alternatively, the attachment of sensors involved the animal being anesthetized (for sensors to be implanted, e.g., Aureli et al., 1999; Bliss-Moreau et al., 2013) or a jacket-system to be fixated (Derakhchan et al., 2014; Kremer et al., 2011) which, if done for the sole purpose of conducting the study, raises ethical concerns and excludes the specially protected great ape species. Additionally, there is evidence that exposure to anesthesia can have short- and long-term influences on cognitive performance both in humans (Caza et al., 2008; Chen et al., 2001; Ing et al., 2012; Rasmussen, 2006) and nonhuman primates (Raper et al., 2015; Talpos et al., 2019; Walters et al., 2019). Results obtained in cognitive tasks following anesthesia thus need to be interpreted with caution. Hence, the past requirement for physical contact of sensors with the participant’s skin has tremendously limited the applicability of cardiac measures in comparative psychological research.

This limited applicability is especially unfortunate since cardiac measures have the potential to provide a unique window into the emotional and cognitive world of nonhuman primates and other species. That is, heart rate signals could be operationalized to measure attentional and affective states in animals. For example, they could function as indicators of primates’ feelings in certain situations or in response to different stimuli. Also, they could complement behavioral measures such as looking-time in studies creating expectation violations to shed light on how nonhuman primates and other animals expect certain social or physical scenes to proceed [(Bräuer & Call, 2011; Horschler et al., 2019; Santos et al., 2005; Uller et al., 1997), for a review see (Winters et al., 2015)]. As with human infants, heart rate measures could also be used to test recognition memory, and therefore help reveal unobservable mental processes in various contexts. This would not only complement existing efforts to understand the evolution of cognition but would also enable cognitive testing of populations and groups of individuals that otherwise do not engage in classic cognitive tasks, such as very young or untrained individuals. Thus, making the measurement of cardiac signals in animals contact-free and with that more applicable would be highly beneficial for comparative psychology advancement.

Developing non-invasive, contact-free ways of measuring cardiac signals will not only open up new ways to get insights into the psychological processes of nonhuman primates and other animals but would also greatly enhance the health management of captive animals. Cardiovascular disease is among the main causes of mortality in captive great apes (Murphy et al., 2018). One of the reasons for this is that monitoring cardiac signals of these potentially dangerous animals is inherently difficult and usually only possible during anesthesia. As a result, signs of cardiac disease, such as abnormal heart rhythms (Lowenstine et al., 2016), are often only recognized very late when the disease has already progressed. The possibility to monitor the cardiac signals of great apes in a new, contact-free way, on a regular basis (perhaps as part of a daily routine), would help to recognize potential signs of cardiac disease earlier and might, therefore, help to lower great ape mortality linked to cardiac disease.

In recent years, the first attempts were made to monitor cardiac signals of nonhuman primates in a contact-free way. Unakafov et al. (2018) designed a non-contact pulse-monitoring system for rhesus macaques. Heart rate was measured by extracting the imaging photoplethysmography (iPPG) signal from a RGB (red, green, and blue) facial video. Although iPPG technology is increasingly used in remote physiological parameter detection for humans, it is limited in estimating cardiac signals for animals with intensely hair-covered skin or thicker epidermal layers. Moreover, the monkeys were restrained and head-stabilized during the study, limiting the value of the method for non-invasive cardiac signal assessment; also see (Froesel, 2020) for a recent study using contact-free HR estimation based on RGB videos and infrared videos with head-restrained rhesus macaques. In a recent pilot study (Al-Naji et al., 2019), a camera-based long-distance monitoring system was explored for extracting the cardiopulmonary signal (heart rate and breathing rate) of diverse exotic animals. The technique relied on the image motion-based method (Al-Naji, Gibson, et al., 2017a; Al-Naji, Gibson, et al., 2017b) rather than the skin color variation used by iPPG technology (Ming-Zher Poh, 2010; Wim Verkruysse, 2008). The motion-based method extracts the cardiopulmonary signal by exploiting the cyclic motion of the animal’s body due to the cardiopulmonary activity. The cardiopulmonary signal was extracted successfully from unrestrained and awake animals, including primates like the Sumatran orangutan (*Pongo abelii*) and hamadryas baboon (*Papio hamadryas*). This technique can be applied on complex surface textures to estimate pulse rate, and it may thus be useful for nonhuman primate studies. While the results of this study appeared extremely promising, they still lack validation by conventional methods.

Here, we demonstrate a non-contact, non-invasive, and real-time monitoring system to extract the heart rate of one of the most commonly tested nonhuman primate species in comparative psychological research, which is also amongst the great ape species most affected by cardiovascular disease: chimpanzees (*Pan paniscus*). In the first study, we validate our monitoring system by means of PPG sensors that were attached to the pointing finger of trained chimpanzees. In a second study, we demonstrate an application of the method as a means to assess the emotional reaction of chimpanzees to different stimuli.

## Study 1

In this study, we validated our monitoring system by means of PPG sensors that were attached to the index finger of trained chimpanzees.

### Methods

#### Animal and ethical considerations

The project was conducted at the Wolfgang Koehler Primate Research Center (WKPRC) in Leipzig, Germany. Chimpanzees were tested individually in their sleeping rooms. The chimpanzees were never food or water-deprived and participated in the study on a voluntary basis. The study was approved by the ethics committee of the Max Planck Institute for Evolutionary Anthropology and Leipzig Zoo. No medical, toxicological, or neurobiological research of any kind is conducted at the WKPRC. All research strictly adheres to the legal requirements of Germany. Animal husbandry and research at the WKPRC comply with the “EAZA Minimum Standards for the Accommodation and Care of Animals in Zoos and Aquaria”, the “WAZA Ethical Guidelines for the Conduct of Research on Animals by Zoos and Aquariums” and the “Guidelines for the Treatment of Animals in Behavioral Research and Teaching” of the Association for the Study of Animal Behavior (ASAB). Prior to data collection, all individuals were trained to extend their index finger through a small hole in the panel and be comfortable with the caregivers attaching a clip to the fingertip.

#### Setup and data acquisition

**Fig. 1:**
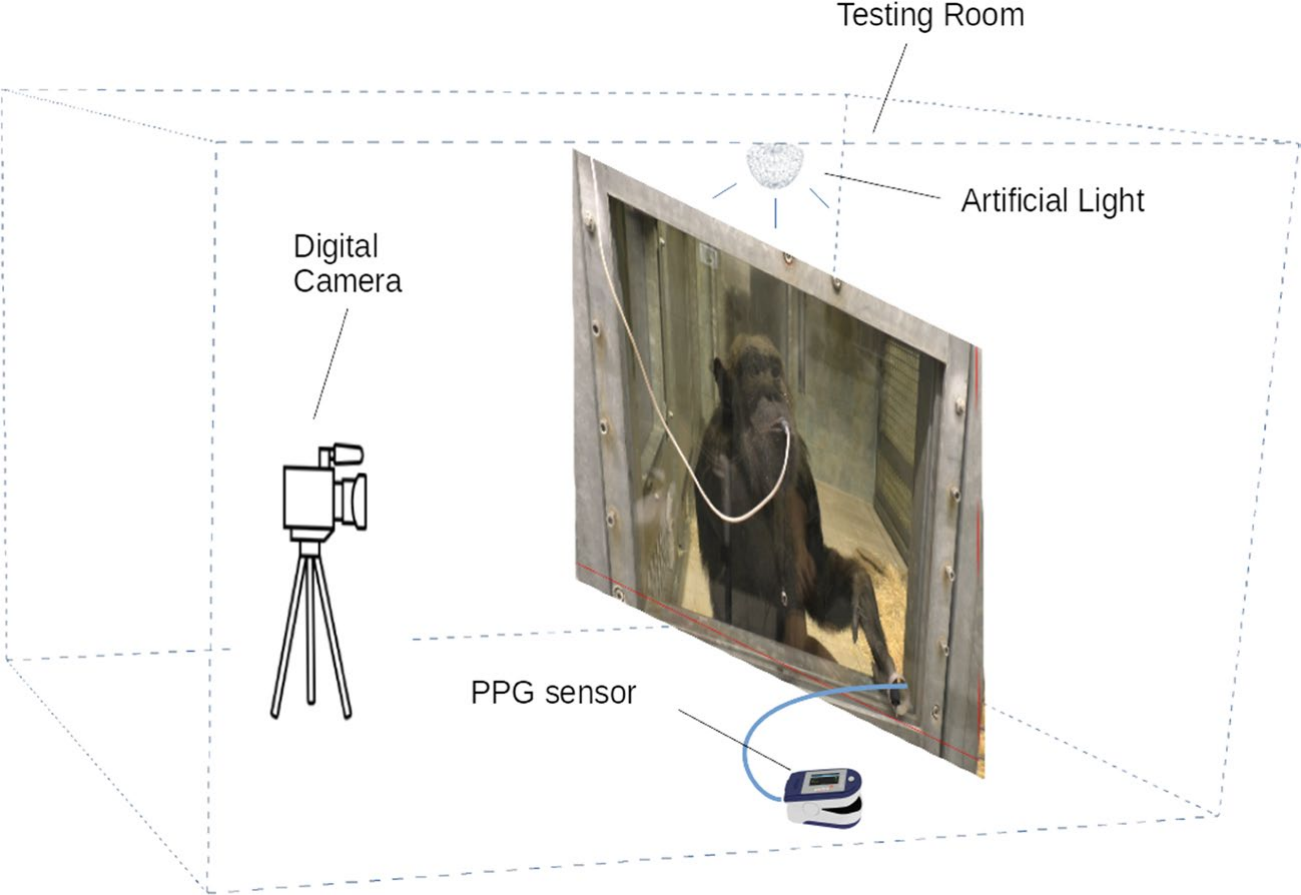
Setup and data collection

The study was performed in an indoor environment (the chimpanzees’ sleeping room), where a digital camera (Panasonic HC-V757 24.0 Mega Pixels) on a tripod was placed at a distance of between 0.5 and 1 m from the front of the chimpanzees’ enclosure, as shown in Fig. 1. To reduce chimpanzee movement, the videos were recorded while the chimpanzees were drinking juice out of a custom-made juice dispenser. At the same time, the chimpanzees extended their fingers through the hole in the Plexiglas to measure the heartbeat by a finger contact PPG sensor (BIOPAC MP160 with PPGED, Pulse range, 30–250 bpm) as the reference heart rate.

Seven chimpanzees (*Pan troglodytes verus*) participated in the study (Table 1). Each testing session lasted up to 10 min, depending on the willingness of the chimpanzee to sit still. Each chimpanzee was tested for up to two sessions, depending on their availability and willingness to participate. For illumination, we used artificial light during video recordings and there was no daylight in the sleeping rooms. The video data were captured with a resolution of 1280 × 720 and a frame rate of 25 fps, saved in MP4 format.

**Table 1:** Chimpanzee data and illumination conditions during the study

Animal	Age (years)	Weight (kg)	Illumination
Alex	19	52.2	Fluorescent
Hope	30	54.9	Fluorescent
Zira	23	48	Fluorescent
Dorien	40	50	Fluorescent
Fraukje	44	42.9	Fluorescent
Sandra	27	51.1	Fluorescent
Shanga	9	35.6	Fluorescent

#### System framework and signal processing

**Fig. 2:**
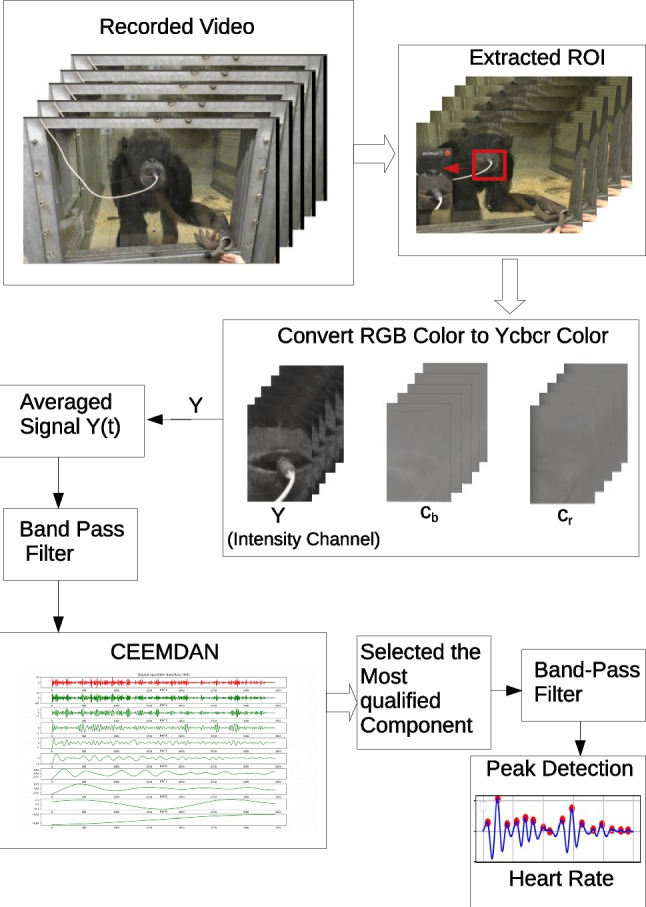
System overview

**Fig. 3:**
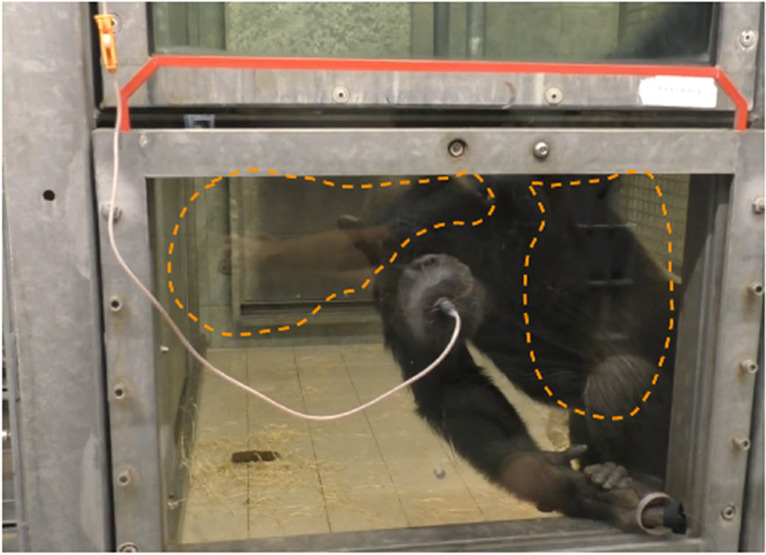
Reflections on the window

The flowchart of the proposed system to compute cardiac signal from videos is presented in Fig. 2. First, we fed the video frame by frame into the system for processing. Once the first frame was captured, the ROI for extracting cardiac signal was manually selected as a bounding-box. Due to the strong reflections caused by the light on the Plexiglas panel (see the areas outlined with the orange dashed line in Fig. 3), we chose the facial area to be the ROI to avoid the noise from reflections (Fig. 2 ).

Cardiopulmonary activity can be extracted from subtle motion information of the body, which is caused by the mechanical flow of blood and the respiratory system (Al-Naji et al. 2017c). These subtle movements directly cause the reflected intensity change in the video. Accordingly, in the second step, since the videos were captured in RGB color space, the intensity information needed to be separated from the color information. The RGB color space was converted to *YC*_*b*_*C*_*r*_ color space using the color space transformation matrix:

1 $$\left[\begin{array}{c}Y\\ {}{C}_b\\ {}{C}_r\end{array}\right]=\left[\begin{array}{ccc}65.841& 128.553& 24.966\\ {}-39.797& -74.203& 112\\ {}112& -93.786& -18.2214\end{array}\right]\left[\begin{array}{c}R\\ {}G\\ {}B\end{array}\right]+\left[\begin{array}{c}16\\ {}128\\ {}128\end{array}\right]$$

R, G, B refer to the pixel values from the RGB color space. We split the *YC*_*b*_*C*_*r*_ frames into three single-channel images and used the intensity channel information to compute the cardiac signal. Then we averaged the intensity pixel values of frame sequences from the ROI with:

2 $${i}_Y(t)=\frac{\sum_{x, y\epsilon ROI}I\left(x,y,t\right)}{\left| ROI\right|}$$

where *I*(*x*, *y*, *t*) is the intensity pixel value at image location (*x*, *y*) at time *t* from recorded frames, and |*ROI*|is the size of the detected ROI that was rectangular.

**Fig. 4:**
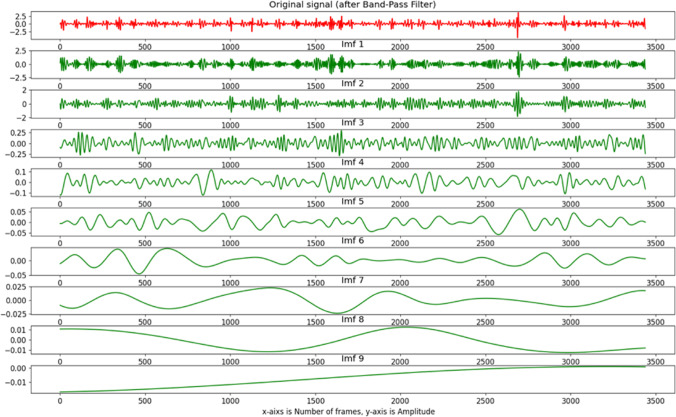
An example of CEEMDAN decomposition of the Y-channel signal in the detected ROI

**Fig. 5:**
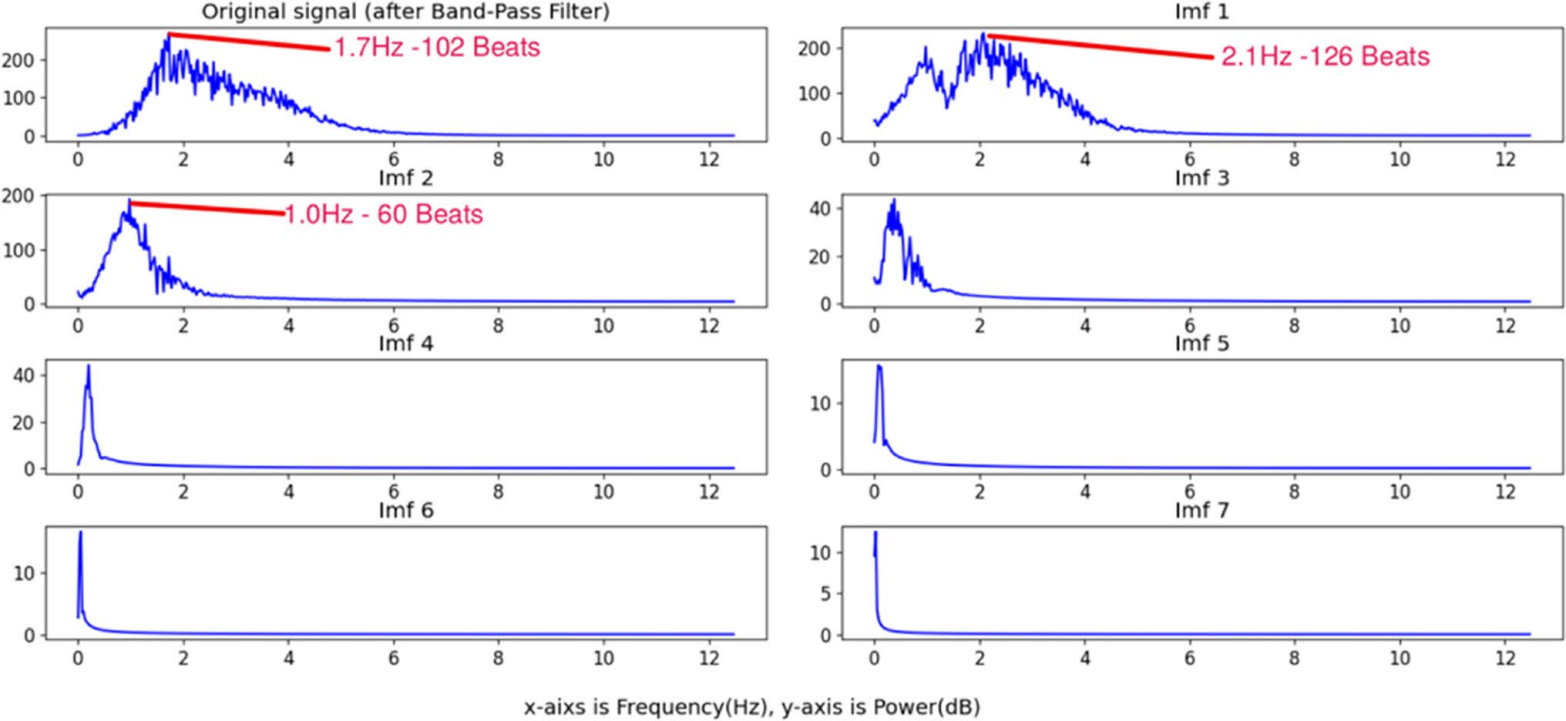
The frequency spectrum of decomposed IMFs (intrinsic mode functions)

The third step is the most important process in the proposed system, which is signal processing. Breathing and cardiac activity both cause subtle body movements with oscillations of different frequencies. We use a band-pass filter (Butterworth coefficients (third-order)) to distinguish between the low-frequency breathing signal (0.25–0.8 Hz corresponding to 15–48 breaths per minute) and the high-frequency cardiac signal (1.5–4.2 Hz corresponding to 90–250 beats per minute). So, after bandpass filtering (1.5–4.2 Hz) of the raw signal extracted from the intensity channel, a complete ensemble empirical mode decomposition with adaptive noise (Colominas et al., 2014) was used to reduce noise interference caused by illumination variations from the raw signal. CEEMDAN decomposes the original signal into intrinsic mode functions (IMF s) with instantaneous amplitude and frequency data. An example of nine IMF decomposition of the Y signals after band-pass filter is provided in Fig. 4. The first mode (IMF 1) after separating the original signal was chosen for estimating the cardiac signal based on the frequency range that corresponds to the typical heartbeat range of chimpanzees (Kearns et al., 2000; Snyder, 1984; Weissler et al., 1961) as shown in Fig. 5.

In the fourth step of the process, a peak detection method was carried out to identify the periodicity of peaks, their locations, and number of peaks of the acquired signals. We computed the heart rate using the following equation,

3 $$Heart\ rate=60\frac{p{F}_r}{n}$$

where *p* is the number of peaks in the acquired signal, *n* is the number of frames of the selected video segment, and *F*_*r*_ is the frame rate.

#### Image processing for ROI stabilization

To reduce false positives of ROI due to target position change and movement during detection, we provide a stabilization method which uses MOSSE Tracker (Bolme et al., 2010) to stabilize the target in every frame. The MOSSE tracker minimizes the sum of squared error between the actual output of the correlation and the desired output of the correlation. We chose it because of its speed. After drawing a bounding-box for tracking target, a MOSSE filter was initialized using the following formula,

4 $${H}^{\ast }=\frac{\sum_i{G}_i\bigodot {F}_i^{\ast }}{\sum_i{F}_i\bigodot {F}_i^{\ast }}$$

where *H*^∗^ is a complex conjugate of the filter. *F*_*i*_ represents the pre-processed cropped template for training images in the Fourier domain for the *i*^*th*^ frame of the video. *G*_*i*_ is the training output image in the Fourier domain for the *i*^*th*^ frame of the video. ${F}_i^{\ast }$ is the complex conjugate of *F*_*i*_. Element-wise multiplication is denoted by the symbol ⨀.

After initialization, the MOSSE filter updates the tracking target from the *i*^*th*^ frame is computed as:

5 $${H}^{\ast }=\frac{A_i}{B_i}$$

6 $${A}_i=\eta {G}_i\odot {F}_i^{\ast }+\left(1-\eta \right){A}_{i-1}$$

7 $${B}_i=\eta {F}_i\odot {F}_i^{\ast }+\left(1-\eta \right){B}_{i-1}$$

where *η* equals to 0.125 as the learning rate to let the filter perform best (Bolme et al., 2010).

**Fig. 6:**
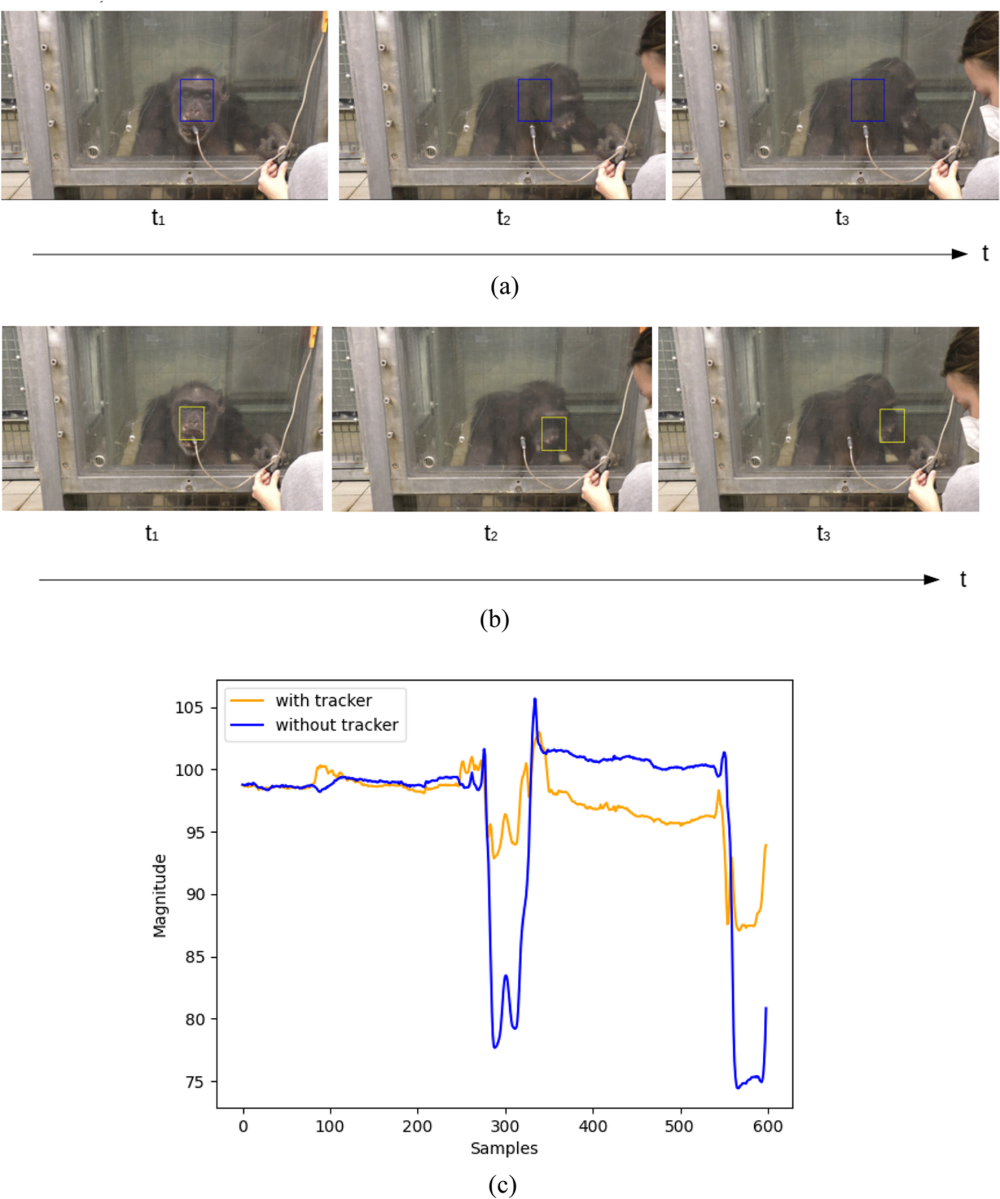
**a** Signals from a video processed without stabilization. **b** Signals from a video with using MOSSE Tracker to stabilize target. **c** Plot of raw signals from a and b

Figure 6 is the comparison of using MOSSE Tracker to track ROI and without tracker for processing a video. During long-term monitoring, when the chimpanzee moved like in Fig. 6a, b. Figure 6b shows that one easily loses the target when a chimpanzee moves their head away at time *t*_1_ and *t*_2_ while the ROI (blue bounding-box) is not being tracked. Figure 6b depicts the same video sequence as in Fig. 6a but using the stabilization method. It is visible that the ROI (yellow bounding-box) is following the head rotation. The raw signals from these two processes are shown in Fig. 6c. It is obvious that the signal (yellow line) processed with the tracker performs better.

### Results

**Fig. 7:**
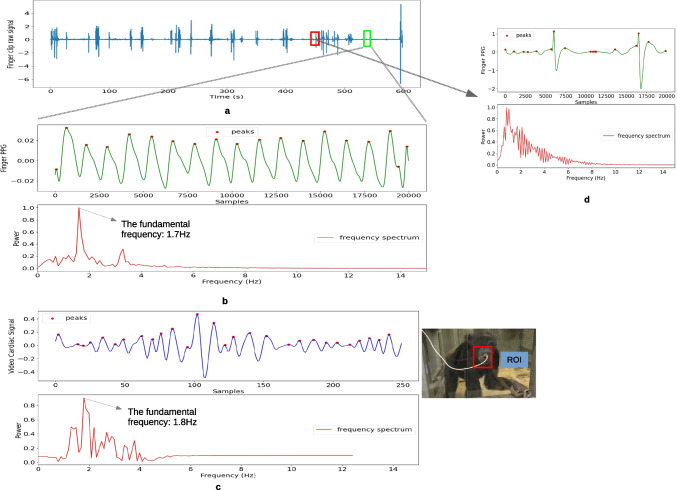
Data comparison of Hope for the same time segment (550–560 s). **a** Raw data of the PPG finger sensor. **b** Smoothed PPG signal and its frequency spectrum. **c** The cardiac signal extracted from video and the frequency spectrum of the signal from video. **d** A noise part of the PPG finger sensor

In this section, we estimated the pulse rate from video based on the motion of the body surface and compared the motion-based pulse rate (video PR) with the reference pulse rate from the finger-contact pulse sensor (PPG sensor). The outcome of our method for measuring heart rate is in agreement with the ground truth heart rate from the pulse sensor. As an example, Fig. 7 shows the results of the video signal and PPG signal from participant Hope. The raw signal from the PPG sensor is shown in Fig. 7a, which is over a total duration of around 10 min (600 s). In the finger sensor raw signal data, it is apparent that there is a lot of noise caused by movement during the period and also probably due to the thick and intensely pigmented skin of chimpanzee fingers. Therefore, a relatively clean data segment (550–560 s) was chosen to compare with the video data. Figure 7b is the smoothed signal from the data segment and the frequency spectrum. Because the video and reference data were recorded synchronously, we selected the video data from the same time period (550–560 s). After band-pass filtering at 1.5 to 4.2 Hz, and separating the signal using CEEMDAN, the cardiac signal extracted from video and its frequency spectrum are shown in Fig. 7c. For the frequency domain of the reference data and the video data, the fundamental frequency shows very close values in pulse rate (1.7 Hz in frequency domain equal to 102 beats per minute and 1.8 Hz in frequency domain equal to 108 beats per minute, respectively). Figure 7d shows the time series of an obvious noise segment (time period 460–480 s in Fig. 7a) and its frequency domain. In Fig. 7d, the error peaks can be easily found in the time domain and the frequency spectrum shows an invalid pulse rate (0.5 Hz).

To validate the usefulness of the motion-based cardiac signal, several quality metrics for video heart rate and contact PPG sensor heart rate are shown in Table 2.

**Table 2:** Quality metrics for pulse rate estimates. Mean error was calculated as the difference between videoHR (video heart rate) and refHR (reference heart rate); the Pearson correlation coefficient was computed between videoHR and refHR across a number of heart rate measurements in each session. The *p* value was calculated for comparing the interpeak distributions of the PPG finger data and our method

Session	Animal	ROI	Time length (s)	Ref-HR (beats/min)	Video-HR (beats/min)	Mean error (%)	KS-test *p* value	Correlation
1	Dorien	Narrow face	210	156.2	153.1	1.98	0.98	0.52
2	Fraukje	Narrow face	240	160.5	169.3	5.48	0.98	0.61
3	Sandra	Narrow face	160	158.7	146.3	7.81	0.99	0.61
4	Dorien	Upper face/ forehead	200	153.1	152.7	0.26	0.98	0.77
5	Fraukje	Upper face/ forehead	240	158.0	159.8	1.14	0.73	0.50
6	Sandra	Upper face/ forehead	260	147.5	141.5	4.07	0.99	0.51
7	Shanga	Upper face/ forehead	100	147.6	144.6	2.03	0.98	0.63
8	Alex	Mouth	130	152.5	146.3	4.07	0.73	0.74
9	Hope	Mouth	110	155.6	153.8	1.16	0.73	0.88
10	Zira	Mouth	200	142.3	150.9	6.04	0.98	0.57

There are ten sessions with different ROIs (narrow face, upper face/forehead, and mouth) which depended on the posture and location of the chimpanzee in the recorded video (they were constantly in motion). In order to have a suitable sequence of video frames (long enough and without target loss) to compare with the contact PPG sensor data, we excluded the noisy parts of each session and computed analysis over noise-free parts only. In this paper, we use 10 s of video data to calculate an average heart rate, and for each session, it needs at least ten consecutive heart rate values to compare with the finger sensor data. Therefore, our criterion for a noise-free sequence to be included was that it was at least 100 s long, during which no target loss occurred. So, in Table 2, the time length of each session for testing is just around 2–3 min. We computed the average video heart rate and the ground truth values of every session. The results show that the average video heart rates are close to the reference data, with the average of all mean errors of 3.4%.

**Fig. 8:**
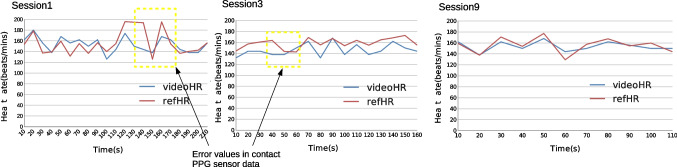
Heart rate values for Sessions 1, 3, and 9, respectively. The heartbeat estimated from the video signal is depicted in *blue*, and the *red color* depicts the heartbeat measured by the finger sensor. The *yellow dash line boxes* show the obviously unstable part of PPG sensor data

**Fig. 9:**
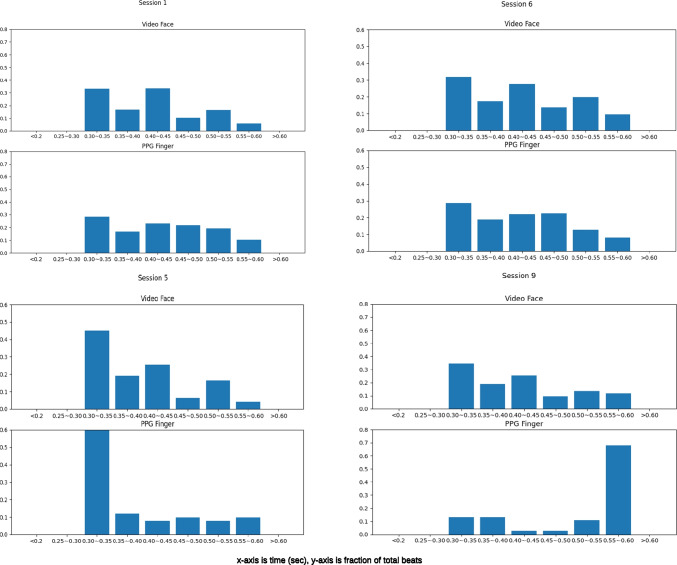
Histograms of four pairs of the beat distributions

The quality of the PPG signal from the PPG sensor might be sub-optimal due to the fact that it is designed based on human skin and, as we explained above, the skin of a chimpanzee’s finger is much thicker than human skin and the texture is rougher as well. Because of this, it is plausible that in Table 2 the correlation values of some sessions (Session 1, 5, and 6) are just around 0.5 but the average value for the overall data (from video and finger contact PPG sensor) are very close. Also, in Fig. 8, which plots the heartbeat from video and finger clip (Sessions 1, 3, and 9), we can see that the finger clip seems to have performed worse, producing unrealistic data. Some very obvious error values have been circled out in a yellow dash line box (normally the heartbeat would not rise or fall rapidly in such a short period of time). The values of the video heartbeat in comparison are rather stable. Thus, due to the unstable performance of the finger clip data, we added the Kolmogorov–Smirnov (KS) test to evaluate the ability of the video cardiac signal to capture subtle heart rate variability (Balakrishnan, 2013). In addition, the final heart rate value is calculated by peak detection (see Eq. 3), so incorrect or missing peaks may produce spurious peak-to-peak intervals, which caused errors in the heart rate. Comparing the distributions of time between successive peaks for each signal might be a better way to validate our method. The KS-test *p* value in Table 2 presents the distribution comparison results; all testing sessions have been accounted for by only considering noise-free intervals with a length within 25% of the average detected pulse period. From the *p* value, we can see that all pairs of distributions are similar. We present four pairs of beat distributions finger clips and videos from Session 1, 5, 6, and 9 in Fig. 9 (an example for different beat distribution shapes: Sessions 1 and 6 show the flat distribution; Sessions 5 and 9 show the peakier distribution). Our method performed a more stable capture capability compared to the data from the finger sensor in all the time ranges.

Because of the unstable performance of the finger sensor on the chimpanzees’ fingers, we also tested our method on humans with the same PPG sensor. We took videos 90–150 s long from three participants and measured the heart rate every 10 s in the same way as with the chimpanzees above. Results are shown in Table 3. The heart rate values are stable from the PPG sensor and the Pearson correlations are round 0.8 to 0.9, which are better than for the chimps (see supplementary material Fig. S1–Fig. S3).

**Table 3:** Comparison of the results of videos and finger sensors from humans

Sub.	ROI	Time Length (s)	Mean absolute error	Correlation	AveHR_Ref (beat/mins)	AveHR_Video (beat/mins)
1	Face	90	1.38	0.84	77	78.4
2	Face	140	0.73	0.87	66.5	67.4
3	Face	100	5.5	0.94	108.1	102.6

### Discussion

Our results show that a novel technique using signals extracted from digital cameras is suitable for measuring heart rate from the faces of chimpanzees. Figure 8 shows that the heart rate accuracy of the technique and the PPG sensor data are not in perfect agreement. We believe that this is mostly due to the fact that the contact PPG sensor was not entirely suitable for chimpanzee skin, rather than due to inaccuracy of our technique.

In this study, the measured pulse rates were in the range of 100–200 beats per minute (BPM) for the seven chimpanzees, with average individual values between 141.5 and 169.3 BMP. These values are within the range of previously reported chimpanzee heart rates 79–240 BPM (Berntson et al., 1989; Boysen & Berntson, 1989; Erickson & Olsen, 1985; Hyeroba, 2011; Kearns et al., 2000; Snyder, 1984; Weissler et al., 1961). Due to the previous difficulty of measuring the heart rate of chimpanzees, the so-far-published heart rate data stem either from awake infant chimpanzees or from anesthetized adult chimpanzees. Expected heart rates of awake adult chimpanzees should range right in between those of anesthetized adult chimpanzees (which were rather low, e.g., 79–91 BPM in Erickson & Olsen, 1985) and awake infant chimpanzees (which were rather high, e.g., 105–240 in Weissler et al., 1961). Our reported average heart rates fulfill this expectation and are thus likely to represent a realistic heart rate range of awake adult chimpanzees. We calculated the heartbeat by counting peaks from every acquired signal. A time window of 10–30 s is required to estimate accurate results.

Since all of the videos were recorded during feeding to reduce movement, long-term observations where the chimpanzee is moving at will could raise some additional challenges. As an alternative means, pulse rate can be estimated from the peak-to-peak interval, which relies heavily on the quality of the signal but might require only a few seconds. In addition, from the *p* values in Table 2 and Fig. 9, it is apparent that our method performed better than the finger sensor in capturing beat-to-beat interval time, which means that the method has great potential to measure heart rate variability accurately. Future work will explore suitable algorithms to define the systolic and diastolic peaks in the extracted signal, and then detect the presence of the shape in the signal.

This study is the extension of research in “A Pilot Study for Estimating the Cardiopulmonary Signals of Diverse Exotic Animals Using a Digital Camera” (Al-Naji et al., 2019). Based on the previous method, the major improvements are: (1) we introduce CEEMDAN (Colominas et al., 2014) to reduce the illumination variations in signal processing part; (2) a stabilization method based on MOSSE Tracker has been used to reduce the noise caused by movements and also to avoid losing the target in the ROI; (3) for the goal of long-term monitoring, the system is running with the possibility of real-time heart rate detection; (4) we validated our method with controlled data from chimpanzees. The results suggest that our study should be suitable for all non-human primates.

## Study 2

In the second study, we demonstrate an application of the method to assess chimpanzees’ emotional reactions to different video stimuli.

### Methods and procedures

#### Animal and ethical considerations

The same seven individuals participated as in Study 1.

#### Setup and data acquisition

**Fig. 10:**
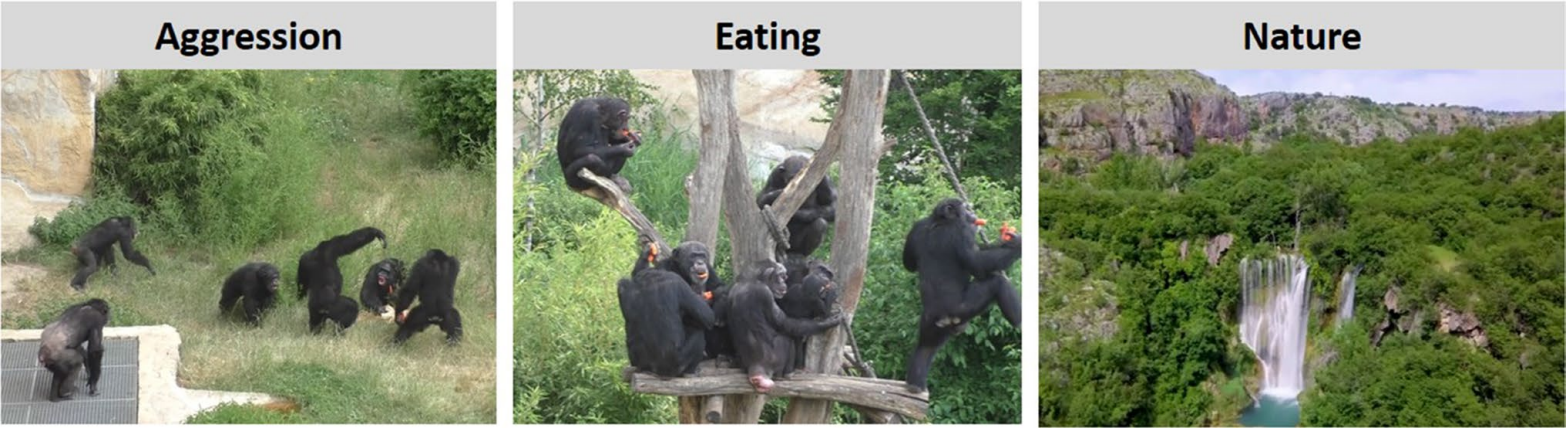
Example images of the three types of videos shown to the chimpanzees. In the aggression condition, familiar conspecifics were engaged in an aggressive interaction. In the eating condition, unfamiliar individuals consumed large amounts of food. In the nature condition, natural habitats were filmed from the bird’s perspective

The setup was identical to Study 1, except that we did not use a PPG finger sensor. Additionally, we placed a screen (37.5 x 30 cm) in front of the Plexiglas panel on which the chimpanzees were presented with different video scenes. The screen was connected to a laptop and video presentations were managed using the software Teobii (Version 3.3.0). The chimpanzees saw three types of video scenes: In the aggression condition, chimpanzees saw conspecifics of their own social group engaged in an aggressive interaction. In the eating condition, the chimpanzees saw conspecifics of another social group (housed at the same zoo) eating large amounts of food. Finally, in the nature condition, the chimpanzees saw videos of natural habitats captured by a drone (see Fig. 10). Both the aggression and the eating scenes included naturally occurring sounds (e.g., screams in aggression scenes, background noises such as bird song in the eating scenes). The nature videos were accompanied by soft ambient music.

**Fig. 11:**
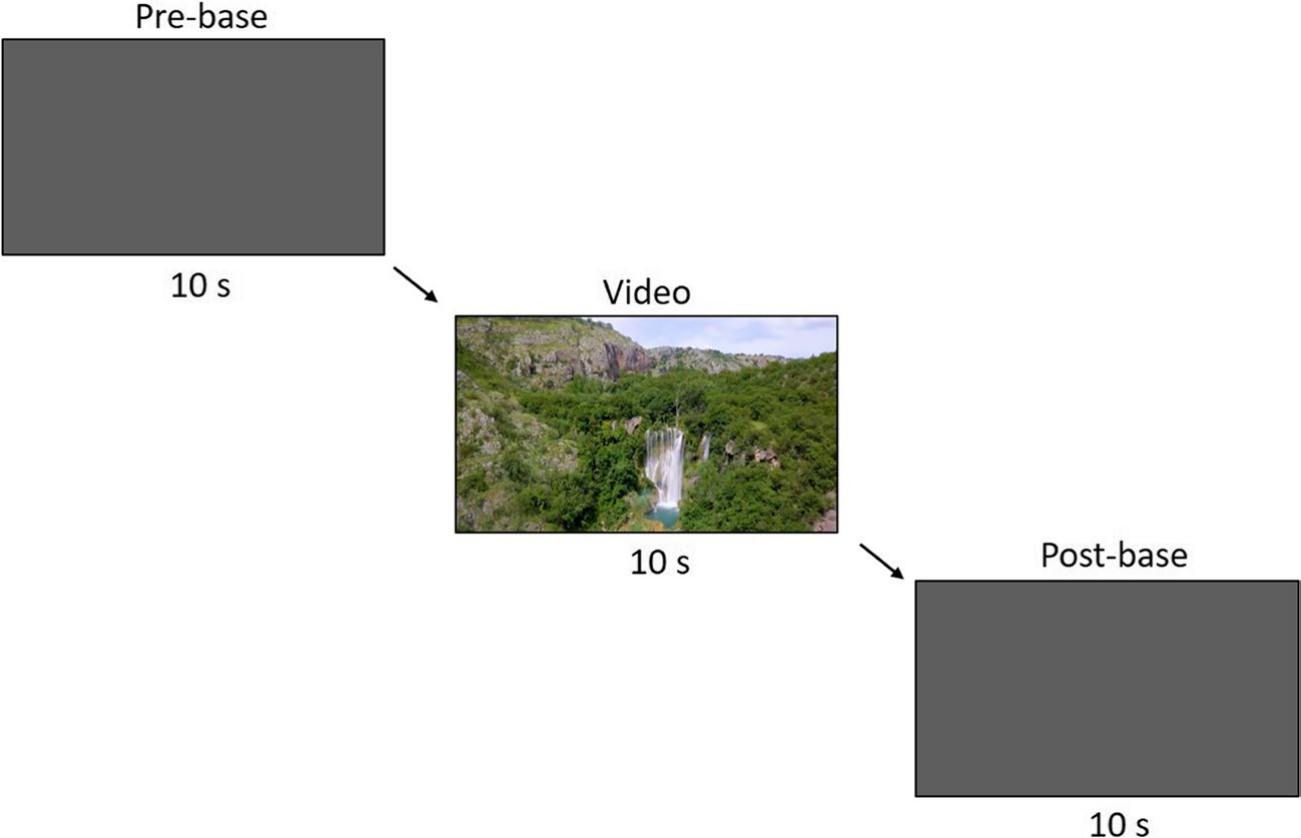
Structure of a trial. Each video presentation was preceded and followed by the presentation of a grey screen during which we measured the chimpanzees’ baseline heart rates

Within one session, the chimpanzees were presented with one trial of each video category. In total, two sessions were administered. That is, the chimpanzees saw two example scenes of each video category (only one chimpanzee, Dorien, saw four examples in total. Her first two sessions were not analyzable due to technical errors and disturbance in the group, respectively). The order of conditions within each session was randomized. Also, the order of example scenes across sessions was randomized (e.g., approximately half of the individuals saw example scene A of the eating condition in their first session, the rest saw it in their second session).

Each video presentation was preceded by the presentation of a grey screen. During this grey-screen presentation, we measured the chimpanzees’ baseline heart rate prior to the video presentation (pre-base). The luminance of the grey screen was identical to that of a representative frame of the respective video. This was to avoid that the chimpanzees’ heart rate changed during video presentation simply due to its visual properties (i.e., its brightness). After the presentation of the video, chimpanzees saw again the same grey screen as before. Here, we recorded the chimpanzees’ heart rate as a post video baseline (post-base). Hence, each trial consisted of three phases: the pre-base, the video, and the post-base. Each of the phases lasted 10 s (see Fig. 11 for an illustration of a trial).

We expected the chimpanzees’ heart rates to change during video presentation relative to the pre-baseline, and to recover during the post-baseline. Further, we predicted the magnitude of change to be influenced by the content of the video: The highest magnitude of change was to be expected in the aggression condition because previous studies have demonstrated that viewing agonistic interactions leads to high levels of arousal in chimpanzees (e.g., Dezecache et al., 2017; Kano et al., 2016). Accordingly, we expected heart rate to increase in this condition. We also expected heart rate to increase during video presentation in the eating condition. This is because we assumed that viewing unfamiliar conspecifics is sufficiently arousing for highly territorial animals, who, in the wild, regularly engage in potentially lethal inter-group interactions (Boesch et al., 2008; Watts et al., 2006; Watts & Mitani, 2001; Wilson & Wrangham, 2003). Similarly, we expected the large amounts of food visible in the eating condition to be potentially arousing to the highly food-motivated animals. In comparison to the aggression condition, however, we expected the relative change in heart rate to be more subtle, mostly because the depicted scene involved fewer indications of potentially arousing affective states. More specifically, while depicted chimpanzees in the eating condition were calmly sitting together, the chimpanzees in the aggression condition uttered screams and showed facial expressions such as fear grins. We expected no change in heart rate during the nature condition because the depicted scenes are supposedly irrelevant to chimpanzees.

### Data analysis

We measured the chimpanzees’ average heart rate during each of the observation phases from video, using the same approach as described for Study 1. Only those trials were included in the analysis where the average heart rate could be calculated for at least pre-base and video phase. Trials in which data for either of these two phases were missing (e.g., because the chimpanzee turned their head away), were excluded. In total, we analyzed 40 trials.

To test if the relative change of heart rate from pre-baseline to video presentation was different depending on which video category the ape had seen, we built a linear mixed model (Bates et al., 2015) with Gaussian error distribution. Average heart rate change (from pre-base to video) was our response variable. As fixed effects, we included condition (eating vs. aggression vs. nature) and session number (within subject, either 1, 2, 3, or 4). Subject was included as random effect on condition. Session was *z*-transformed (to a mean of 0 and a standard deviation of 1). To keep type 1 error rate to the nominal level of 5% (Barr, 2013; Schielzeth & Forstmeier, 2009), we included all possible random slopes components (condition and session number within subject) and the respective correlations between random slopes and intercepts. The significance of the full model as compared to the null model (comprising only session and the random effect subject) was established using a likelihood-ratio test (R function ANOVA with argument test set to ’Chisq’; (Dobson & Barnett, 2008; Forstmeier & Schielzeth, 2011)). *P* values for the individual effects were obtained using the package ’lmerTest’ (Kuznetsova et al., 2017).

To check if, within each of the three conditions, heart rate was different during the three trial phases, we ran three linear mixed models with Gaussian error distribution, one for each condition. Average heart rate was the response variable. Phase (pre-base, video, post-base), as well as (*z*-transformed) session were included as fixed effects. Subject was included as random effect on phase. We included all possible random slopes components (phase and session number within subject) and the respective correlations between random slopes and intercepts. The significance of the full model as compared to the null model (comprising only session and the random effect subject was established using a likelihood-ratio test (R function ANOVA with argument test set to “Chisq”; (Dobson & Barnett, 2008; Forstmeier & Schielzeth, 2011)). *P* values for the individual effects were based on likelihood-ratio tests comparing the full model with respective reduced models (R function drop1). All models were fitted in R (R Core Team, 2021) using the function lmer of the R-package lme4 (Bates et al., 2015). Raw data and analysis code can be found in the supplementary material.

### Results

**Fig. 12:**
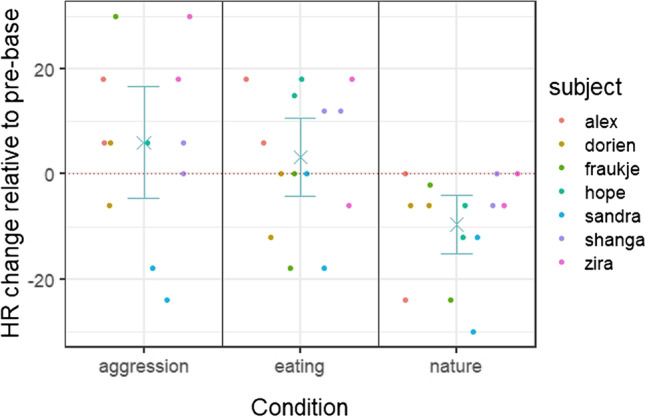
Heart rate change during video presentation relative to the pre-baseline in the three conditions. *Dots* represent individual data per session for each chimpanzee. *Dashed line* represents pre-baseline level. *Turquoise stars* depict average changes over all individuals and sessions with bootstrapped 95% confidence intervals

We found a highly significant effect of condition (i.e., video category) on the relative HR change from pre-baseline to video presentation (*X*^2^ = 9.91, *df* = 2, *p* = 0.002), i.e., heart rate changed differently depending on the video category. As visible in Fig. 12, there was a significant difference in HR change between the nature and the aggression condition (estimate ± SE = – 15.332 ± 3.771, *df* = 2, *p* = 0.001, CI (– 22.723, – 7.942)), with a decrease in heart rate in the nature condition, and an increase in heart rate in the aggression condition. There was also a significant difference in HR change between the nature and the eating condition (estimate ± SE = 12.786 ± 4.169, *df* = 2, *p* = 0.0134, CI (20.957, 4.614)), with an increase in heart rate in the eating condition. There was no difference between the eating and the aggression condition (estimate ± SE = – 2.547 ± 4.339, *df* = 2, *p* = 0.570, CI (– 11.051, 5.957)), but Fig. 12 illustrates that the heart rate change was slightly more pronounced in the aggression condition. There was no effect of session number (estimate ± SE = 0.454 ± 3.246; *df* = 1; *p* = 0.896).

Looking at the three conditions separately, we found a significant effect of phase on heart rate in the nature condition (*X*^2^= 7.436, *df* = 2, *p* = 0.01817). More specifically, heart rate decreased significantly during the video presentation compared to the pre-baseline (estimate ± SE= – 9.571 ± 2.695, *df* = 2, *p* = 0.009, CI (– 15.661, – 3.482)) and was also significantly lower than in the post-baseline (estimate ± SE = – 9.619 ± 4.14, *df* = 2, *p* = 0.047, CI ± (0.611, 18.973); see Fig. 12 and Fig S.7 in the supplementary material). There was no significant effect of phase in either the eating condition (*X*^2^ = 0.440, *df* = 2, *p* = 0.3818) or the aggression condition (*X*^2^ = 2.363, *df* = 2, *p* = 0.3432). Individual data for each session are visualized in the supplementary material (Fig. S 4–Fig. S 6).

### Discussion

In this study, we measured the heart rate of chimpanzees in response to viewing different categories of social and non-social videos. We found that heart rate changed in response to the video playbacks. The direction and magnitude of change were dependent on the content of the video. As expected, heart rate increased during both the aggression condition and the eating condition (although absolute levels of heart rate were not significantly different from the pre- and post-baseline in both cases). While heart rate accelerated slightly more in the aggression condition than in the eating condition, this difference was not significant, suggesting that both feeding conspecifics from a foreign group and agonistic interactions of group mates had an arousing effect on chimpanzees. These findings align with previous results using different measures of emotional arousal (Dezecache et al., 2017; Kano et al., 2016).

Interestingly, and contrasting our predictions, we found a significant decrease in heart rate during the presentation of nature videos. There are several possible explanations for this finding.

The first possible explanation for the chimpanzees’ decelerated heart rate in the nature condition is that the nature stimuli may have induced the orienting response, i.e., an increase in attention with low levels of emotional arousal.

When human infants orient towards and attend to a (typically novel) stimulus, they enter a phase of “sustained attention” in which their heart rate is lower compared to baseline levels. Heart rate only resumes to baseline levels once the infant terminates attention (e.gColombo, 2001; Lansink, 1997; Richards, 1992). This particular change of heart rate is part of the so-called orienting response, which presumably subserves enhanced stimulus detection and processing (e.g., Graham & Clifton, 1966; Stekelenburg, 2002).

There is some evidence for a similar effect in chimpanzee infants (Berntson, 1984; Berntson, 1989; Berntson et al., 1989). In these studies, chimpanzee infants were presented with auditory stimuli, such as conspecific screams and conspecific laughter (Berntson et al., 1989), or threat, stress, and alarm vocalizations (Berntson, 1989) or with a combination of auditory and tactile stimuli (a tone and a vibration, Berntson, 1984). The infant chimpanzees’ heart rates decelerated when they listened to screams (Berntson et al., 1989) and, upon initial presentation, also when they listened to threat, stress, and alarm vocalizations (but upon repeated exposure, the authors report heart rate acceleration in response to the same stimuli, Berntson, 1989). Conversely, chimpanzee infants’ heart rate accelerated when they listened to conspecific laughter (Berntson et al., 1989), and upon first exposure to the tone and the tactile stimulus (but upon repeated exposure, the authors report heart rate deceleration in response to the same stimuli, Berntson, 1984). One study also assessed juvenile chimpanzees’ heart rates in response to visual stimuli (Boysen & Berntson, 1989). Here it was found that heart rate decelerated in response to seeing pictures of an unfamiliar chimpanzee, whereas it accelerated in response to seeing pictures of an aggressive chimpanzee. In sum, previous research found heart rate deceleration in response to hearing negatively valanced stimuli (aggression and stress) and seeing neutral foreign conspecifics.

In contrast to these previous studies, the chimpanzees’ heart rates in our study accelerated in response to the videos depicting aggressive interactions, which were also accompanied by threat vocalizations and screams (which is in accordance with other, more recent studies showing increased emotional arousal in response to such displays, e.g., Dezecache et al., 2017; Kano et al., 2016). Also, it increased in response to seeing unfamiliar conspecifics. These differences could be either due to the difference in modality (auditory or static picture stimuli in previous studies vs. video (visual + auditory) stimuli in our study), or to differences in the tested age class (infant and juvenile chimpanzees in previous studies vs. adult chimpanzees in our study), or to differences in the method used to measure heart rate. Currently, it remains unclear if adult chimpanzees show signatures of the orienting response when presented with dynamic visual stimuli and when heart rate is measured with state-of-the-art technology.

However, the finding that chimpanzees’ heart rate decelerated in the nature condition in our study could be interpreted as being indicative of the chimpanzees displaying the orienting response in this condition. Presumably, all our videos attracted the apes’ attention, which should, according to the theory of the orienting response, result in a temporarily decelerated heart rate. The videos of conspecifics, however, may have additionally induced emotional arousal, leading to overall higher heart rates compared to baseline levels. The videos of natural landscapes, by contrast, may have not induced emotional arousal but still induced sustained attention, leading to overall lower heart rates relative to baseline levels. It is noteworthy that both explanations for the decelerated heart rate in the nature condition are not mutually exclusive. It is well possible that the nature stimuli, in contrast to the conspecific stimuli, activated both the orienting response and had a calming effect on the chimpanzees.

Future research should use a method with higher temporal resolution to investigate if the typical heart rate changes associated with the orienting response can be found in adult chimpanzees and under which conditions stimuli induce the orienting response rather than emotional arousal/relaxation.

A second possible explanation for the decelerated heart rate in the nature condition is that viewing these stimuli had a relaxing effect on chimpanzees. Such a calming effect might have been caused by the perceptual features of these stimuli. More specifically, the nature scenes involved slowly and regularly moving patterns, whereas the other scenes, especially the aggression scene, involved irregularly and fast-moving elements. Moreover, in contrast to the two other conditions, the nature scenes were accompanied by soft, ambient music. This type of music is known to have a relaxing effect on humans (e.g., Bernardi et al., 2006; Chlan, 1998; Hilz et al., 2014; Krumhansl, 1997; Nomura et al., 2013) and may hence also cause a decrease in heart rate in other apes. It is possible that either the ambient music, or the perceptual properties of the nature scene, or a combination of both, had a relaxing effect on the chimpanzees, irrespective of the depicted content.

Alternatively, the content of the stimuli, i.e., the depicted nature scenes themselves, may have had a relaxing effect on the apes. This would be unexpected because the video scenes were drone footage of different natural habitats shot from a bird’s perspective. That is, they depicted views entirely unfamiliar to our chimpanzees.

Studies on humans have found that spending time in nature, or viewing nature-related stimuli, does have a relaxing effect on humans, indicated, among others, by decelerated heart rate (Brown et al., 2013; Park et al., 2010; Sahlin et al., 2016). The finding that our closest living relatives may react similarly to natural stimuli they have never encountered in real life could suggest that this relaxing effect of nature does not depend on previous positive experiences in the depicted habitats. Instead, the positive, calm feeling many humans associate with spending time in nature may be grounded in an innate physiological response to natural environments deeply rooted in our evolutionary history. Natural environments may be important for our physiological and mental well-being, and the finding that this importance might have an evolutionary origin could emphasize the value of nature conservation. Assuming that lower resting heart rate is associated with lower stress levels and better well-being (e.g., Aureli et al., 1999; Sommerfeldt, 2019; Shively, 2015), our results also suggest that cardiac measures might be a promising tool for assessing the welfare of captive chimpanzees and other animals.

When designing enclosures, zoos typically try to ensure that they are suitable for animals to fulfill their primary biological needs and, more recently, naturalistic designs are often favored over artificial optics. One main motivation behind this trend is that visitors value naturalistic enclosures and enrichment over non-naturalistic ones (e.g., Davey, 2006; Kutska, 2009; Melfi et al., 2004); Ogden et al., 1993; Wolf & Tymitz, 1981). Some studies also investigated the benefits of naturalistic enclosures for animal welfare and found that these designs are often associated with the expression of natural behaviors and low frequencies of psychopathologies (e.g., Chang et al., 1999; Fàbregas et al., 2012; Maple & Finlay, 1986; Maple & Stine, 1982). Other studies, however, suggested that it is not a naturalistic appearance that is crucial for animal welfare, but other factors like sufficient space or functional enclosure complexity (e.g., Cassinello & Pieters, 2000; Clubb & Mason, 2003; Clubb & Mason, 2007; Fàbregas et al., 2012; Stroud, 2007). In fact, some authors suggested that the naturalistic appearance of an enclosure is entirely unsuitable for assessing how well it meets the needs of its occupants (Melfi et al., 2004; Shepherdson, 1998). While more systematic research is needed to draw firm conclusions, our study may give a first hint that viewing nature-related stimuli might have positive effects on the well-being of chimpanzees (and possibly other animals). We suggest that future studies should complement traditional welfare measures such as ranking the degree of expressed natural behaviors and psychopathologies (see, e.g., Maple & Perdue, 2013 for a comprehensive review of traditional measures of well-being and well-fare of zoo animals) with contact-free cardiac measures in order to assess an animals’ well-being in a more nuanced and direct manner. Importantly, once the appropriate algorithms have been developed, future research should also consider an animal’s heart rate variability (HRV), i.e., the variability of the time intervals between consecutive heartbeats. HRV is indicative of the functioning of the autonomic nervous system, in particular between sympathetic and vagal activity, and therefore gives a more comprehensive and detailed picture of an individual’s physiological and psychological state compared to heart rate alone (e.g., McCraty, 1995; Sleigh & Henderson, 1995). HRV is regularly used as a marker of acute and chronic stress in humans (Delaney & Brodie, 2000; Hall et al., 2004). Also in animals, HRV is often assessed to analyze changes in sympathovagal balance related to diseases, psychological and environmental stressors, and welfare (see Von Borell, 2007 for a review). Ideally, future studies should systematically assess and compare the resting heart rate and HRV in individuals of the same species living in different types of enclosures that vary with regard to their level of natural appearance. If such studies confirmed that animals living in more natural enclosures have on average lower resting heart rates and higher HRV, this would suggest that both functional complexity and natural appearance should be considered when designing new enclosures.

## General discussion

In this study, we validated a new method of measuring heart rate in a contact-free way in a nonhuman species. The results show that our technique can estimate the heart rate of conscious and unrestrained chimpanzees without special hardware for illumination. Furthermore, since the input videos can be recorded with a standard digital camera, our technique is affordable and easy to use. It thus has the potential to be applicable in a wide range of contexts and setups.

In addition to validating our method, we have also demonstrated a simple application for comparative psychological research. We obtained findings that have potential implications for the evolutionary origins of the positive effects of nature on human well-being, and for the welfare of zoo-housed apes and other animals. This shows that our technique opens up new routes to study aspects of animals’ emotional and cognitive worlds that have previously been obscured. In the current study, we used contact-free heart rate monitoring to measure emotional arousal in chimpanzees. For future research, it will be interesting to try and measure more subtle changes of emotional states, such as surprise or changes in cognitive processing such as cognitive load. Being able to monitor these states will open up an even wider range of possible applications and research questions. Measuring heart rate in a contact-free way brings many advantages in comparison to traditional techniques. First, it increases the number of animals for psychological research with nonhuman primates. Standard measures that involved attaching one or several sensors to a participant’s body were only applicable with very well trained individuals or individuals who underwent anesthesia before the studies. This constraint severely limited the number of testable individuals, especially for research with great apes. Our technique eliminates these constraints and hence allows for much larger samples which, in turn, lead to more meaningful results of comparative studies. In addition, the contact-free nature of our technique also allows observing groups of individuals who do not readily interact with researchers and hence have rarely been tested in comparative research. For example, young great ape infants who are still clinging to their mothers have been practically inaccessible to psychological research, leaving significant gaps in knowledge regarding their cognitive development. Our technique makes this group of individuals accessible for cognitive testing and allows us to ask new research questions in the area of comparative developmental research.

The second advantage of contact-free heart rate monitoring is that it has the potential to improve the health management of captive animals. More specifically, the contact-free and easy application of the method allows for regular monitoring of animals’ cardiac signals without disturbing the daily routine of the animals and their caretakers. In turn, this may facilitate recognizing potential signs of cardiac disease in the early stages and thereby help lower mortality linked to cardiac disease. Hence, our method is not only beneficial for basic cognitive research but can also greatly enhance the health management of a wide range of animal species.

In order to allow for unattended monitoring of great apes’ heart rate, future research should employ more complex filtering and decomposition algorithms to enhance the robustness of the pulse signal extraction. More specifically, our current method is limited to situations in which animals remain relatively still for at least 5–6 consecutive seconds, such as during feeding or sleeping. Large or sudden movements will cause the tracker to lose the target and signal extraction will be interrupted. More complex filtering and decomposition algorithms will be needed to reduce susceptibility to movement and make it more applicable for a wider range of contexts.

### Supplementary Information

ESM 1 (DOCX 981 kb)

ESM 2 (ODT 10 kb)
