# Supplementary material for: Estimating the cardiac signals of chimpanzees using a digital camera: validation and application of a novel non-invasive method for primate research
Source: Behav Res Methods. 2023 May 30;56(3):2064–82. doi: 10.3758/s13428-023-02136-y (PMC10991041; doi:10.3758/s13428-023-02136-y)
Supplement: Supplementary file 1 — (DOCX 981 kb) [file 13428_2023_2136_MOESM1_ESM.docx]

Supplementary material


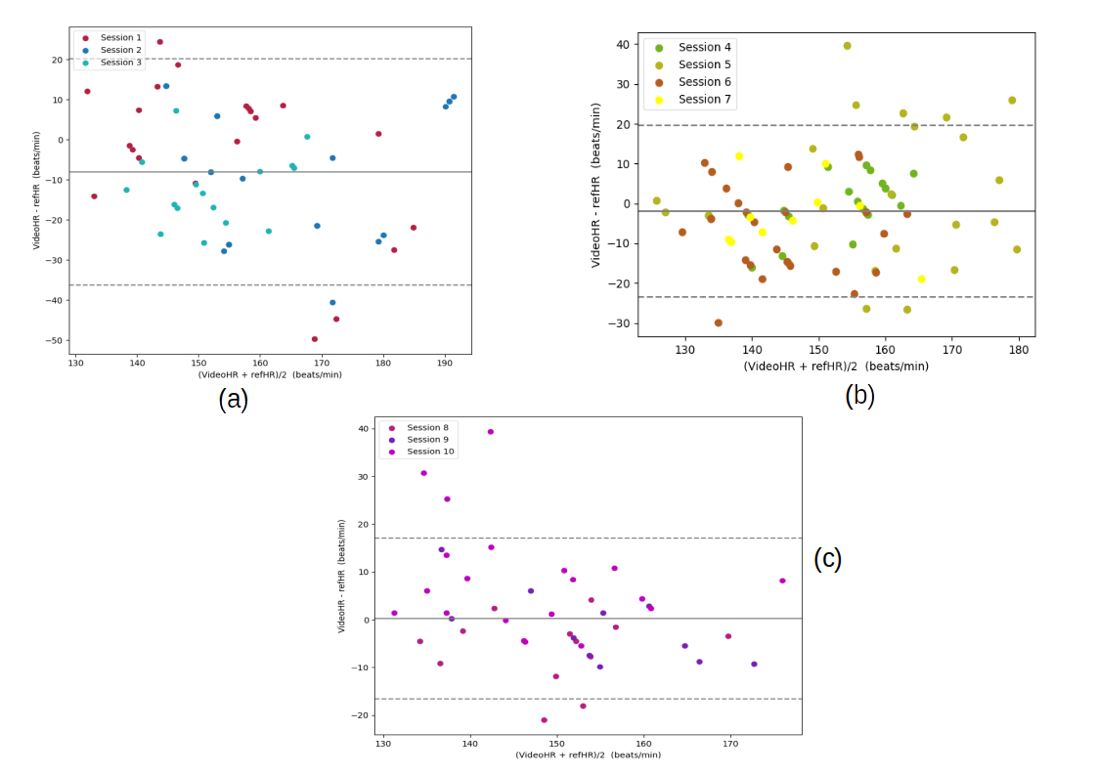


Fig. S 1: Bland-Altman plots of heart rate estimates from chimpanzees. Solid line shows mean error(videoPR-refPR), dashed lines indicate mean error ±1.96 times the standard deviation of the error. (a) Bland-Altman plots of sessions with ROI of narrow face. (b) Bland-Altman plots of sessions with ROI of upper face/forehead. (c) Bland-Altman plots of sessions with ROI of mouth.


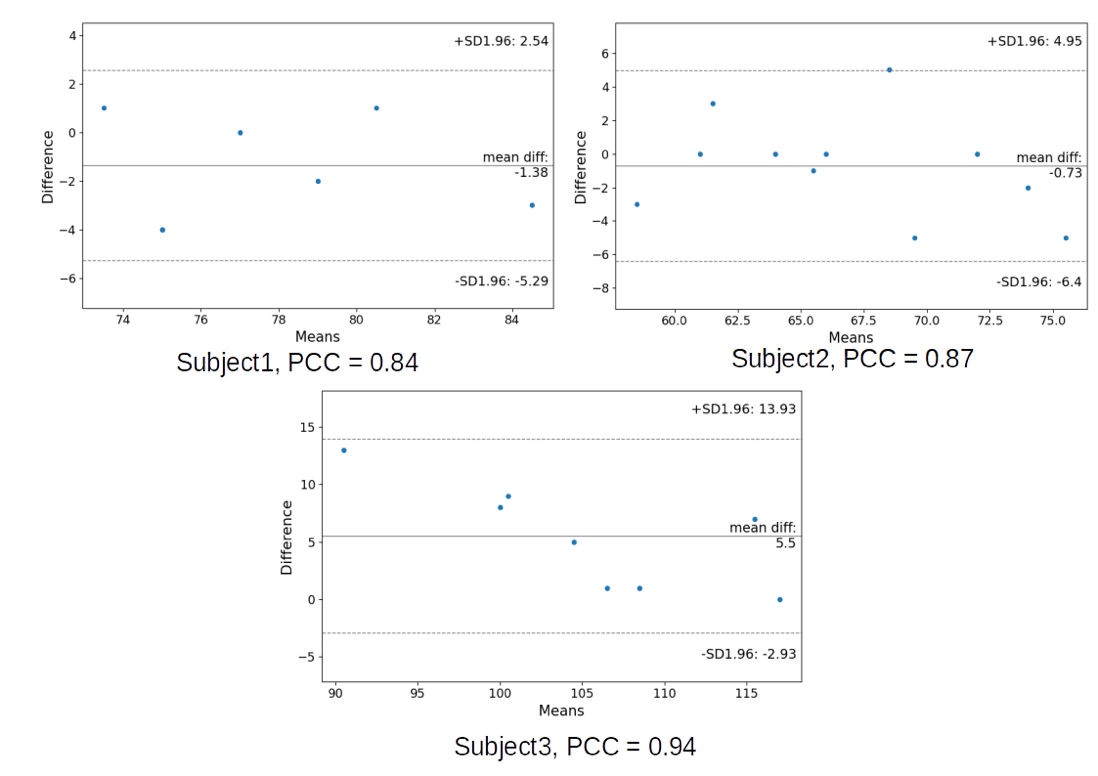


Fig. S 2: Bland-Altman plots of heart rate estimates from humans.


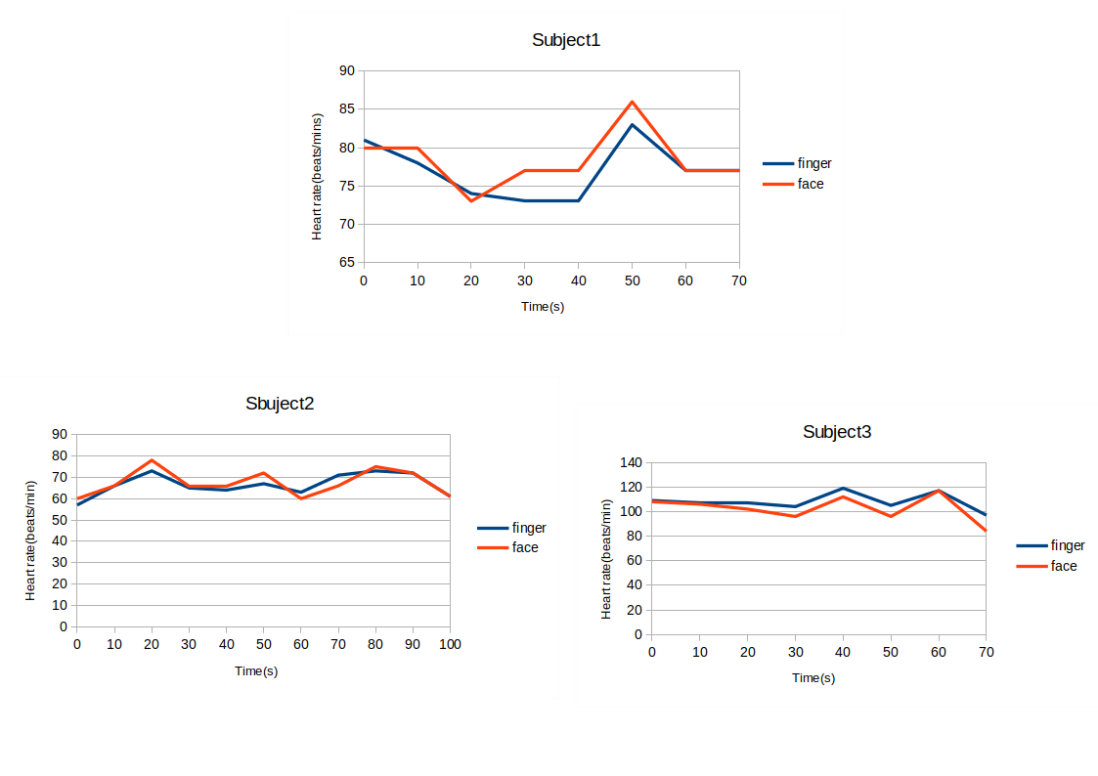


Fig. S 3: Heart Rate values for the three participants respectively. The heartbeat estimated from the video signal is depicted in blue, and the red color depicts the for heartbeat measured by the finger sensor.


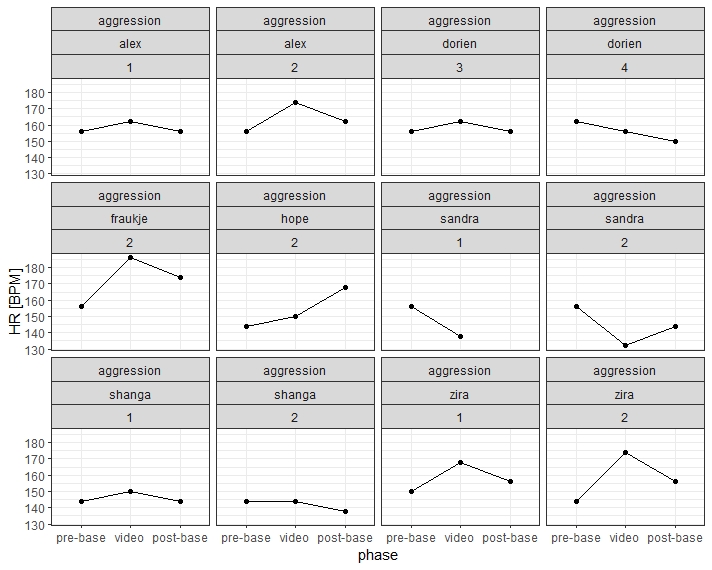


Fig. S 4 : Individual data for aggression session.


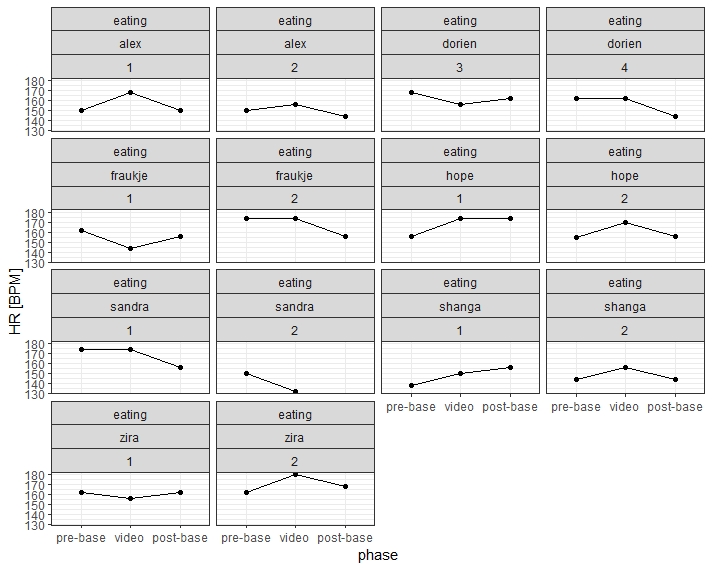


Fig. S 5: Individual data for eating session.


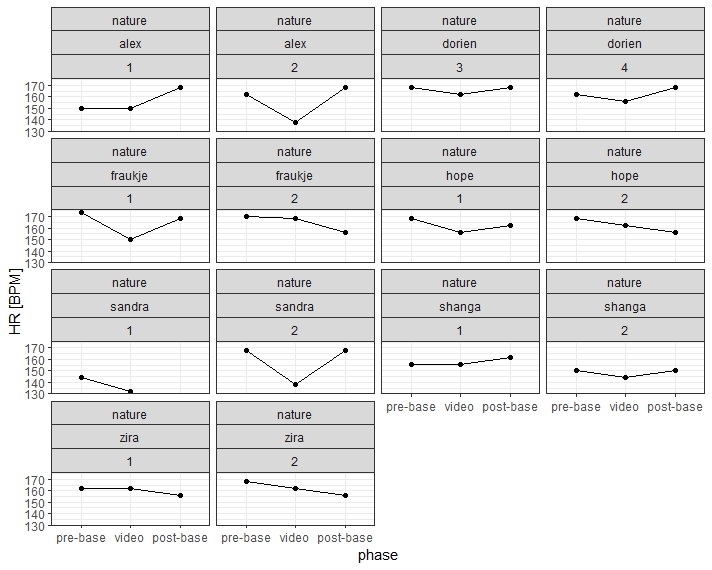


Fig. S 6: Individual data for nature session.


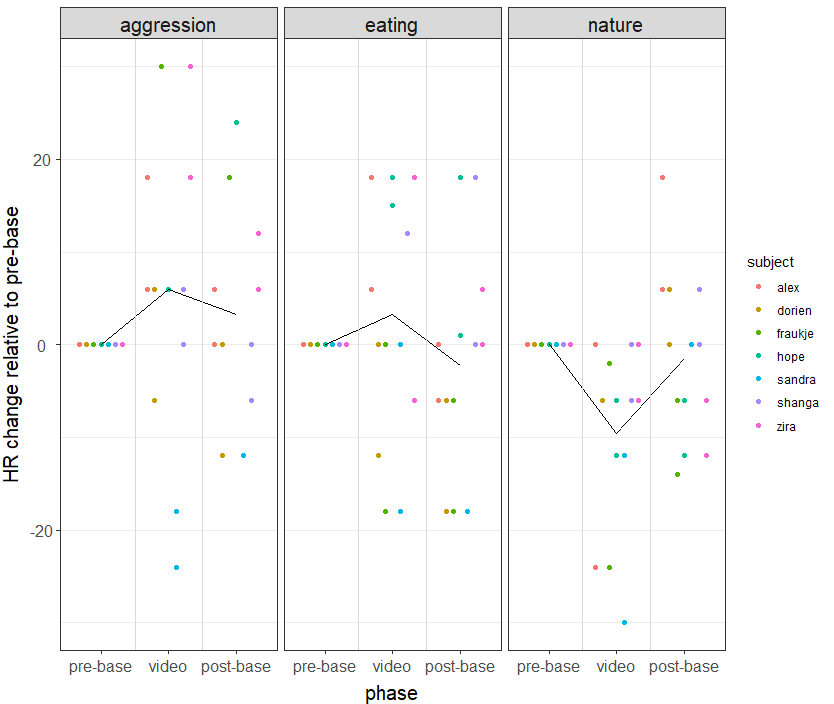


Fig. S7: Average heart rate change relative to the pre-baseline for the video and the post-base line phase of each condition. Dots represent individual data for each chimpanzee and each session. Solid lines depict average changes over all individuals.
