# Supplementary material for: Estimating the cardiac signals of chimpanzees using a digital camera: validation and application of a novel non-invasive method for primate research
Source: Behav Res Methods. 2023 May 30;56(3):2064–82. doi: 10.3758/s13428-023-02136-y (PMC10991041; doi:10.3758/s13428-023-02136-y)
Supplement: Supplementary file 2 [file 13428_2023_2136_MOESM2_ESM.odt]

Supplementary Material

Link for source code:

https://github.com/danni203/caridac_signal_chimp

Link for data and data analyses scripts:

[https://drive.google.com/drive/folders/19laDtSezozDy2H3Aw06RAWb1EYbWEHJj?usp=sharing](https://drive.google.com/drive/folders/19laDtSezozDy2H3Aw06RAWb1EYbWEHJj?usp=sharing)
